# Sampling the Variational Posterior with Local Refinement

**DOI:** 10.3390/e23111475

**Published:** 2021-11-08

**Authors:** Marton Havasi, Jasper Snoek, Dustin Tran, Jonathan Gordon, José Miguel Hernández-Lobato

**Affiliations:** 1 Department of Engineering, University of Cambridge, Cambridge CB2 1PZ, UK; jongordon06@gmail.com (J.G.); jmh233@cam.ac.uk (J.M.H.-L.); 2 Brain Team, Google Research, Mountain View, CA 94043, USA; jsnoek@google.com (J.S.); trandustin@google.com (D.T.)

**Keywords:** bayesian inference, variational inference, deep neural networks, contextual bandits

## Abstract

Variational inference is an optimization-based method for approximating the posterior distribution of the parameters in Bayesian probabilistic models. A key challenge of variational inference is to approximate the posterior with a distribution that is computationally tractable yet sufficiently expressive. We propose a novel method for generating samples from a highly flexible variational approximation. The method starts with a coarse initial approximation and generates samples by refining it in selected, local regions. This allows the samples to capture dependencies and multi-modality in the posterior, even when these are absent from the initial approximation. We demonstrate theoretically that our method always improves the quality of the approximation (as measured by the evidence lower bound). In experiments, our method consistently outperforms recent variational inference methods in terms of log-likelihood and ELBO across three example tasks: the Eight-Schools example (an inference task in a hierarchical model), training a ResNet-20 (Bayesian inference in a large neural network), and the Mushroom task (posterior sampling in a contextual bandit problem).

## 1. Introduction

Uncertainty plays a crucial role in a multitude of machine learning applications, ranging from weather prediction to drug discovery. Poor predictive uncertainty risks potentially poor outcomes, especially in domains such as medical diagnosis or autonomous vehicles, where high confidence errors may be especially costly [1]. Thus, it is tremendously important that the underlying model provides high quality uncertainty estimates along with its predictions. By marginalizing over a posterior distribution over the parameters given the training data, Bayesian inference provides a principled approach to capturing uncertainty. Unfortunately, exact Bayesian inference is not generally tractable. Variational inference (VI) instead approximates the true posterior with a simpler distribution. VI is appealing since it reduces the problem of inference to an optimization problem, where the goal is to minimize the discrepancy between the true posterior and the variational posterior. The key challenge, however, is the task of training expressive posterior approximations that can capture the true posterior without significantly increasing computational and memory costs. The most widely used one is the mean-field approximation, where the posterior is represented using an independent Gaussian distribution over all the model parameters. The mean-field approximation is easy to train, but it fails to capture dependencies and multi-modality in the true posterior.

This paper describes a novel method for generating samples from a highly flexible posterior approximation. The idea is to start with a coarse, mean-field approximation and make a series of inexpensive, local refinements to it. At the end, we draw a sample from the refined region. We show that through this process, we can generate samples that capture both *dependencies* and *multi-modality* in the true posterior.

**Figure 1:**
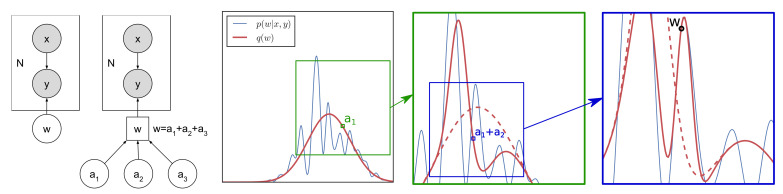
(**Left**) The supervised learning model and augmented model, respectively, where *w* is expressed as a sum of independent auxiliary variables. (**Right**) High level illustration of the refining algorithm. In each iteration, the value of an auxiliary variable is fixed, and the posterior is locally adjusted. In the final iteration, a sample is drawn from q(w). Through the iterations, the variational distribution is able to approximate well the true posterior in a local region.

The refinements take place at gradually decreasing scales starting with large scale changes, moving towards small scale adjustments. The regions of these adjustments are determined by sampling the values of additive auxiliary variables. Formally, we express the model parameters w using a number of additive auxiliary variables $w=a_{1}+\ldots +a_{K}$ (Figure 1 left) that leave the marginal distribution unchanged. The refinement process takes place over *K* optimization steps. In each step, we sample the value of an auxiliary variable according to the current variational approximation $a_{k}∼q\left(a_{k}\right)$ and optimize the approximation by conditioning on the newly sampled value $q\left(w\right)\approx p\left(w|x,y,a_{1:k}\right)$ (k=1…K). At the end, we obtain a sample $w=a_{1}+\ldots +a_{K}$ from the refined posterior $q_{\mathrm{ref}}\left(w\right)$. To obtain further samples, we must go back to our initial, coarse approximation and repeat the *K*-step process again. We refer to the refinements as local, because after sampling each auxiliary variable, the process moves towards smaller scale adjustments until it reaches w.

The refined posterior is a highly flexible approximation to the true posterior. It is able to capture dependencies and multi-modality even when these are absent from the initial variational approximation. We demonstrate the multi-modality of the refined posterior on a synthetic example, and we show how the refined posterior is able to capture dependencies in a hierarchical inference problem.

We theoretically show that the refined posterior improves the ELBO over the initial variational approximation. We also demonstrate this empirically by applying the method to Bayesian neural networks on common regression and image classification benchmarks.

Generating each sample requires a series of optimization steps that come with associated computational costs. We found that in a deep neural network, the computational overhead of generating a small set of samples for prediction amounts to ∼30% of the cost of training the initial variational approximation; thus, the refinement process is able to generate a set of high-quality posterior samples at the cost of a small computational overhead (compared to training a standard mean-field approximation).

An ideal application of our method is using it to generate posterior samples for Thompson sampling, which is a popular approach to tackle contextual bandit tasks. It works by sampling a random hypothesis from the posterior to decide on each action. In this scenario, the computational cost is not a key consideration, we can spend further computation on generating high quality posterior samples. We show that the high quality samples generated by refining the posterior improve the performance of Thompson sampling in contextual bandit task as measured by the cumulative regret.

### Organization of the Paper

In Section 2, we start by introducing the notation and giving an overview of variational inference. Then, we present our proposed algorithm for generating samples from a refined variational distribution. Through two examples, we show that refined posterior can capture both dependencies and multi-modality. In Section 3, we provide theoretical guarantees that the refinement step always improves the quality of the variational distribution (measured by the ELBO) under mild conditions. In Section 4, we evaluate the effectiveness of the method on Bayesian neural networks on a set of UCI regression and image classification benchmarks. We observe that our method consistently improves the quality of the approximation, as evidenced by a higher ELBO and likelihood of the samples. We also demonstrate that the high-quality posterior samples can be used in Thompson sampling to reduce the cumulative regret in a contextual bandit task. In Section 5, we discuss a related works and place our method in context.

## 2. Materials and Methods

In this section, we first describe standard variational inference (VI), followed by a detailed description of our proposed sample generation method that refines the variational posterior. The inputs and labels are denoted by x⊆X and y⊆Y, respectively, and w denotes the model parameters.

### 2.1. Variational Inference

Exact Bayesian inference is often intractable and is NP-hard in the worst case. Variational inference attempts to approximate the true posterior p(w|x,y) with an approximate posterior $q_{\phi }\left(w\right)$, typically from a simple family of distributions, for example independent Gaussians over the weights, i.e., the mean-field approximation. To ensure that the approximate posterior is close to the true posterior, the parameters of $q_{\phi }\left(w\right)$, ϕ are optimized to minimize their Kullback–Leibler divergence: $\mathrm{KL}\left[\;q_{\phi }\left(w\right)\;\;\left|\right|\;\;p\left(w|x,y\right)\;\right]$. At the limit of $\mathrm{KL}\left[\;q_{\phi }\left(w\right)\;\;\left|\right|\;\;p\left(w|x,y\right)\;\right]=0$, the approximate posterior exactly captures the true posterior, although this might not be achievable if p(w|x,y) is outside of the distribution family of $q_{\phi }\left(w\right)$.

In order to minimize the KL-divergence, variational inference optimizes the evidence lower bound (ELBO) w.r.t. ϕ (denoted as L(ϕ)), which is a lower bound to the log marginal likelihood logp(y|x). Since the marginal log-likelihood can be expressed as the sum of the KL-divergence and the ELBO, maximizing the ELBO is equivalent to minimizing the KL divergence:

(1) $$\begin{array}{cc} \log p(y|x) & =\underset{\geq 0}{\underset{︸}{\mathrm{KL}\left[\;q_{\phi }\left(w\right)\;\;\left|\right|\;\;p\left(w|x,y\right)\;\right]}}+L\left(\phi \right)\\ & \geq L(\phi )\\ \; & =E_{q_{\phi }}\left[\log p(y|x,w)\right]-\mathrm{KL}\left[\;q_{\phi }\left(w\right)\;\;\left|\right|\;\;p\left(w\right)\;\right] \end{array}$$

due to non-negativity of the KL-divergence.

Following the optimization of ϕ, the model can be used to make predictions on unseen data. For an input $x^{\prime }$, the predictive distribution $p(y^{\prime }|x^{\prime },y,x)$ can be approximated by stochastically drawing a small number of sample model parameters $w_{1:M}∼q_{\phi }\left(w\right)$ and averaging their prediction in an ensemble model $p\left(y^{\prime }|x^{\prime },y,x\right)\approx \frac{1}{M}\sum_{i=1}^{M}p\left(y^{\prime }|x^{\prime },w_{i}\right)$.

### 2.2. Refining the Variational Posterior

The main issue with variational inference is the inflexibility of the posterior approximation. The most widely used variant of variational inference, mean-field variational inference, approximates the posterior with independent Gaussians across all dimensions. This approximation is too simplistic to capture the complexities of the posterior for complicated models. With our proposed method, it is feasible to generate samples from a detailed posterior by starting with a mean-field approximation and refining it in selected, local regions. Note that the method does not yield an analytic form to the detailed posterior, it generates a set of samples $w_{1:M}$ from it.

The graphical model is augmented with a finite number of auxiliary variables $a_{1:K}$ as shown in Figure 1. The constraints are that (x,y) must be conditionally independent of the auxiliary variables given w and that they must not affect the prior distribution p(w). These constraints ensure that the marginal likelihood logp(y|x) is unchanged, enabling us to train the augmented model with the same ELBO as the unaugmented model; thus, *the model is unaffected by the presence of the auxiliary variables*. Their purpose is solely to aid the inference procedure. Given a Gaussian prior $N(w|0,\sigma_{w}^{2}I)$ over w, we express w as a sum of independent auxiliary variables (Although we are focusing on one specific definition of the auxiliary variables, additive auxiliary variables, note that all of our results straight-forwardly generalize to arbitrary joint distributions $p\left(w,a_{1:K}\right)$ that meet the constraints).

$$w=\sum_{k=1}^{K}a_{k},\;\mathrm{with}\;p\left(a_{k}\right)=N\left(a_{k}|0,\sigma_{a_{k}}^{2}I\right)\;\mathrm{for}\;k=1\ldots K\;,$$

while ensuring that $\sum_{k=1}^{K}\sigma_{a_{k}}^{2}=\sigma_{w}^{2}$, so that the prior $p\left(w\right)=N\left(w|0,\sigma_{w}^{2}I\right)$ remains unchanged.

We refine the approximate posterior to generate each sample $w_{1:M}$. Specifically, this refers to iteratively sampling the values of the auxiliary variables $a_{1:K}$ and then approximating the posterior of w, conditional on the sampled values, i.e., $q_{\phi_{k}}\left(w\right)$ approximates $p\left(w|x,y,a_{1:k}\right)$ for iterations k=1…K ($\phi_{k}$ is dependent on $a_{1:k}$) as shown in Algorithm 1.
**Algorithm 1:** Refine and Sample ($\phi_{0}$)
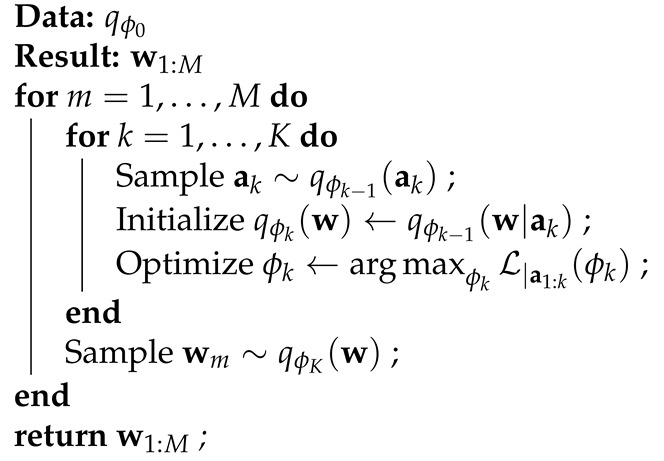


That is, starting from the initial mean-field approximation $q_{\phi_{0}}\left(w\right)$, for k=1,…,K,

1. Sample the value of $a_{k}$ using the current variational approximation and fix its value.
  (2) $$a_{k}∼q_{\phi_{k-1}}\left(a_{k}\right)=\int p\left(a_{k}|a_{1:k-1},w\right)q_{\phi_{k-1}}\left(w\right)\;dw$$
  A sample can be obtained by first sampling $w∼q_{\phi_{k-1}}\left(w\right)$ followed by $a_{k}∼p\left(a_{k}\right|a_{1:k-1},$w). This is straightforward for exponential families and factorized distributions. The closed form for $q_{\phi_{k-1}}\left(a_{k}\right)$ is provided in the Appendix A.
2. Optimize the variational approximation conditional on the sampled $a_{k}$: $q_{\phi_{k}}\left(w\right)\approx p\left(w|x,y,a_{1:k}\right)$.
  (3) $$\phi_{k}\leftarrow arg min\mathrm{KL}\left[\;q_{\phi_{k}}\left(w\right)\;\;\left|\right|\;\;p\left(w|x,y,a_{1:k}\right)\;\right]$$
  This optimization is very fast in practice if $\phi_{k}$ is initialized using the solution from the previous iteration: $q_{\phi_{k}}\left(w\right)\overset{\mathrm{init}}{\leftarrow }q_{\phi_{k-1}}\left(w|a_{k}\right)$. The closed form of $q_{\phi_{k-1}}\left(w|a_{k}\right)$ provided in the Appendix A.

We then obtain $w=\sum_{k=1}^{K}a_{k}$. Analogous to VI, the KL-divergence in step 2 is minimized by maximizing the conditional ELBO

(4) $$L_{|a_{1:k}}\left(\phi_{k}\right)=E_{q_{\phi_{k}}}\left[\log p\left(y|x,w\right)\right]-\mathrm{KL}\left[\;q_{\phi_{k}}\left(w\right)\;\;\left|\right|\;\;p\left(w|a_{1:k}\right)\;\right]\;,$$

where $p\left(w|a_{1:k}\right)=N\left(w|\sum_{i=1}^{k}a_{i},I\left(\sigma_{w}^{2}-\sum_{i=1}^{k}\sigma_{i}^{2}\right)\right)$. Note that, when k=K, the numerical minimization of $\mathrm{KL}\left[\;q_{\phi_{k}}\left(w\right)\;\;\left|\right|\;\;p\left(w|x,y,a_{1:k}\right)\;\right]$ is unnecessary since in this case, the optimal $q_{\phi_{K}}\left(w\right)$ is a delta function located at the sum of the sampled $a_{1:K}$.

In order to generate *M* independent samples $w_{1:M}$ from the refined posterior, the previous process has to be repeated *M* times, sampling new values for $a_{1:K}$ each time.

### 2.3. Multi-Modal Toy Example

**Figure 2:**
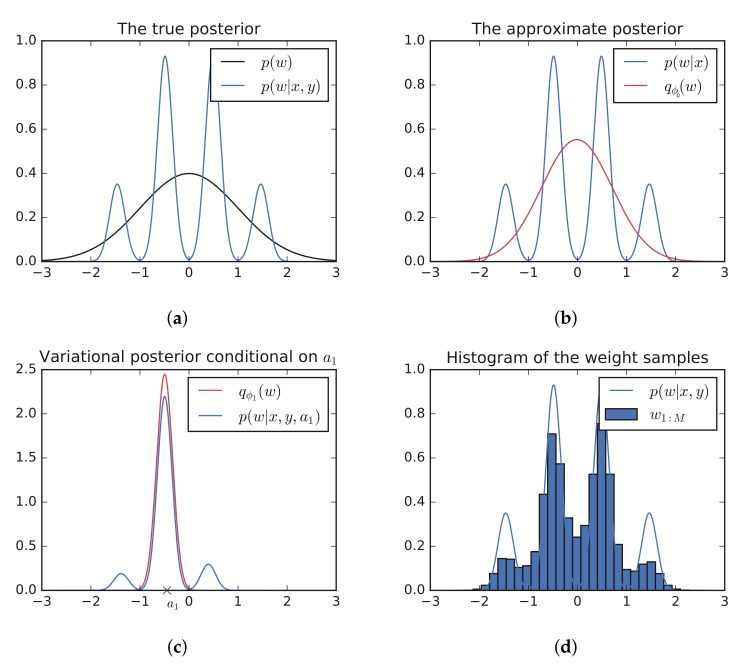
Our method can capture a multi-modal posterior starting with a Gaussian posterior approximation. (**a**) The true posterior, which is too complex to be well approximated by a Gaussian distribution. (**b**) The Gaussian approximate posterior after optimizing the ELBO (ELBO=−1.79). (**c**) We sample $a_{1}$, optimize the resulting conditional ELBO to obtain $q_{\phi_{1}}\left(w\right)$ and then sample $w_{m}∼q_{\phi_{1}}\left(w\right)$. This whole process repeats m=1,…,M times to obtain $w_{1:M}$. (**d**) Histogram of the samples $w_{1:M}$ obtained from the refined posterior approximation. ELBO≥−1.45.

We use a synthetic toy example to demonstrate the procedure and to show that through the refinement steps, the approach is able to capture multiple posterior modes. In this example, we have a single weight w with prior p(w)=N(w|0,1) and a complex posterior with four modes. Figure 2b shows that a Gaussian approximation fails to capture the multi-modal nature of the true posterior.

We express w as the sum of K=2 auxiliary variables: $w=a_{1}+a_{2}$ with $p\left(a_{1}\right)=N\left(a_{1}|0,0.8\right)$ and $p\left(a_{2}\right)=N\left(a_{2}|0,0.2\right)$, which recovers p(w)=N(w|0,1) as per the constraint. The first step of the refinement process is sampling $a_{1}∼q_{\phi_{0}}\left(a_{1}\right)=\int p\left(a_{1}|w\right)q_{\phi_{0}}\left(w\right)\;dw$, where $q_{\phi_{0}}\left(w\right)$ is an initial mean field approximation to the posterior. Then, the variational posterior is optimized conditional on the sampled $a_{1}$; that is, $\phi_{1}=arg min\mathrm{KL}\left[\;q_{\phi_{1}}\left(w\right)\;\;\left|\right|\;\;p\left(w|x,y,a_{1}\right)\;\right]$. Figure 2c shows that the conditional variational posterior is able to fit one of the posterior modes. Over many runs, the different values of $a_{1}$ force the conditional posterior to fit different posterior modes, thus allowing the refined posterior to capture the multi-modal nature of the true posterior as shown in Figure 2d. Clearly, the refined posterior is a much better approximation to the true posterior than the Gaussian approximation though we note that the true posterior is not recovered exactly.

### 2.4. Capturing Dependencies: A Hierarchical Example

In this section, we use the eight-schools example from STAN [2,3] to show how the refined posterior can capture dependencies among the hidden variables and to discuss the effect of the number of auxiliary variables on the quality of the posterior approximation.

The eight-schools example studies the coaching effect of 8 schools. Each school reports the mean $y_{i}$ and standard error $\sigma_{i}$ of its coaching effect where i=1,…,8. There is no prior reason to believe that any school was more effective than another so the model is stated in a hierarchical manner:

$$µ∼N\left(0,25\right),\;\;\;\;\;\tau ∼\mathrm{HalfCauchy}\left(0,5\right),\;\;\;\;\;\theta_{i}∼N\left(µ,\tau^{2}\right),\;\;\;\;\;y_{i}∼N\left(\theta_{i},\sigma_{i}^{2}\right)\;\;\mathrm{for}\;i=1\ldots 8\;,$$

where the HalfCauchy distribution refers to a Cauchy distribution supported only on positive values (i.e., a symmetric half of the Cauchy distribution).

**Figure 3:**
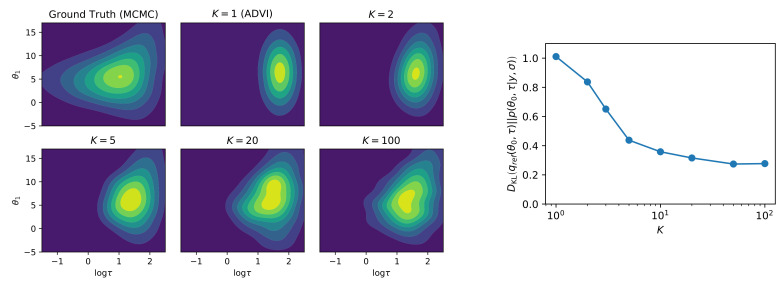
(**Left**) The refined posterior for increasing numbers of auxiliary variables. As *K* increases, the refined posterior is able to capture the dependency between $\theta_{1}$ and τ. (**Right**) The KL divergence between the refined posterior and approximate posterior decreases as *K* grows. (Calculated using kernel density estimation.)

Factorized approximations perform poorly on this problem due to the dependency of θ on τ (for an excellent analysis of this problem, see [4]). In fact, the MAP solution is at τ=0, which is distant from the mean-field approximation that STAN uses for variational inference (ADVI, [5]) (Figure 3 left).

We show that our method can capture the dependencies between θ and τ. We introduce the following additive auxiliary variables:

$$\begin{array}{c} µ=\sum_{k=1}^{K}a_{{µ}_{k}}\;a_{{µ}_{k}}∼N\left(0,\frac{25}{K}\right),\;\tau =|\sum_{k=1}^{K}a_{\tau_{k}}|\;\\ a_{\tau_{k}}∼\mathrm{Cauchy}\left(0,\frac{5}{K}\right),\;\theta =µ+\tau \sum_{k=1}^{K}a_{\theta_{k}}\;a_{\theta_{k}}∼N\left(0,\frac{1}{K}\right), \end{array}$$

for k=1…K. As required by the constraints, the auxiliary variables leave the model unchanged.

Figure 3 left shows the approximate posterior for various *K* values. At K=1, the model is equivalent to ADVI, and as *K* increases, we can see that the refined posterior is able to capture the dependencies between τ and $\theta_{1}$ and results in a non-Gaussian form. The ground truth samples were obtained using the NUTS sampler in PyMC3 [6,7]. The density plots were generated using kernel-density-estimation.

### 2.5. Limit as K→∞

A natural question to ask is what happens as the number of auxiliary variables grows to infinity. We can estimate the KL-divergence of the refined posterior and the true posterior in the eight-schools example using kernel density estimation based on the samples generated from the refined posterior. We see that it monotonically decreases (Figure 3 middle). Indeed, we show theoretically that each auxiliary variable increases the ELBO and hence decreases the KL-divergence to the true posterior. However, the precise condition for convergence to the true posterior remains an open question.

## 3. Theoretical Results

We claim that the refinement process must improve the variational approximation over the initial mean-field approximation as measured by the ELBO.

This claim is formalized in the following proposition.

**Proposition** **1.** *Let*

$${\mathrm{ELBO}}_{\mathrm{ref}}=\underset{q_{\mathrm{ref}}}{E}\left[\log p\left(y|x,w\right)\right]-\mathrm{KL}\left[\;q_{\mathrm{ref}}\left(w\right)\;\;\left|\right|\;\;p\left(w\right)\;\right]$$

*be the ELBO of the refined posterior (where $q_{\mathrm{ref}}$ is the distribution that our process generates samples from), let*

$$\begin{array}{c} {\mathrm{ELBO}}_{\mathrm{aux}}=\underset{q_{\mathrm{ref}}}{E}\left[\log p\left(y|x,w\right)\right]-\mathrm{KL}\left[\;q_{\mathrm{ref}}\left(a_{1:K}\right)\;\;\left|\right|\;\;p\left(a_{1:K}\right)\;\right] \end{array}$$

*be the ELBO accounting for the auxiliary variables, and let*

$${\mathrm{ELBO}}_{\mathrm{init}}=\underset{q_{\phi_{0}}}{E}\left[\log p\left(y|x,w\right)\right]-\mathrm{KL}\left[\;q_{\phi_{0}}\left(w\right)\;\;\left|\right|\;\;p\left(w\right)\;\right]$$

*be the ELBO of the initial variational approximation. Then, the following inequalities hold:*

$${\mathrm{ELBO}}_{\mathrm{ref}}\geq {\mathrm{ELBO}}_{\mathrm{aux}}\geq {\mathrm{ELBO}}_{\mathrm{init}}.$$

Thus, ${\mathrm{ELBO}}_{\mathrm{ref}}$, the ELBO of the distribution that we are generating samples from is greater than, or equal to ${\mathrm{ELBO}}_{\mathrm{init}}$, the ELBO of the initial mean-field approximation.

### 3.1. Proof of ${\mathrm{ELBO}}_{\mathrm{ref}}\geq {\mathrm{ELBO}}_{\mathrm{aux}}$

This is a consequence of the fact that $a_{1:K}$ fully determines w.

**Proof.**  $$\begin{array}{cc} {\mathrm{ELBO}}_{\mathrm{ref}}-{\mathrm{ELBO}}_{\mathrm{aux}}\; & =\mathrm{KL}\left[\;q_{\mathrm{ref}}\left(a_{1:K}\right)\;\;\left|\right|\;\;p\left(a_{1:K}\right)\;\right]-\mathrm{KL}\left[\;q_{\mathrm{ref}}\left(w\right)\;\;\left|\right|\;\;p\left(w\right)\;\right]\\ \; & =\underset{q_{\mathrm{ref}}\left(a_{1:K}\right)}{E}\left[\log\frac{q_{\mathrm{ref}}\left(a_{1:K}\right)}{p\left(a_{1:K}\right)}-\log\frac{q_{\mathrm{ref}}\left(w\right)}{p\left(w\right)}\right]\\ \; & =\underset{q_{\mathrm{ref}}\left(w\right)}{E}\left[\underset{q_{\mathrm{ref}}\left(a_{1:K}|w\right)}{E}\left[\log\frac{q_{\mathrm{ref}}\left(a_{1:K}\right)}{p\left(a_{1:K}\right)}-\log\frac{q_{\mathrm{ref}}\left(w\right)}{p\left(w\right)}\right]\right]\\ \; & =\underset{q_{\mathrm{ref}}\left(w\right)}{E}\left[\underset{q_{\mathrm{ref}}\left(a_{1:K}|w\right)}{E}\left[\log\frac{q_{\mathrm{ref}}\left(a_{1:K}|w\right)}{p\left(a_{1:K}|w\right)}\right]\right]\\ \; & =\underset{q_{\mathrm{ref}}\left(w\right)}{E}\left[\underset{\geq 0}{\underset{︸}{\mathrm{KL}\left[\;q_{\mathrm{ref}}\left(a_{1:K}|w\right)\;\;\left|\right|\;\;p\left(a_{1:K}|w\right)\;\right]}}\right]\geq 0\;, \end{array}$$

where line 4 follows using Bayes’ theorem: $q_{\mathrm{ref}}\left(a_{1:K}|w\right)=\frac{q_{\mathrm{ref}}\left(w|a_{1:K}\right)q_{\mathrm{ref}}\left(a_{1:K}\right)}{q_{\mathrm{ref}}\left(w\right)}$, $p\left(a_{1:K}|w\right)=\frac{p\left(w|a_{1:K}\right)p\left(a_{1:K}\right)}{p\left(w\right)}$ and that $q_{\mathrm{ref}}\left(w|a_{1:K}\right)=p\left(w|a_{1:K}\right)=\delta_{Dirac}\left(w-\sum_{k=1}^{K}a_{k}\right)$. The proof is concluded using the non-negativity of the KL-divergence. □

Note that ${\mathrm{ELBO}}_{\mathrm{ref}}$ is a valid ELBO—it is a lower bound to the marginal likelihood $\log p\left(y|x\right)\geq {\mathrm{ELBO}}_{\mathrm{ref}}$. Therefore, optimizing ${\mathrm{ELBO}}_{\mathrm{ref}}$ through our sampling procedure decreases the KL divergence between $q_{\mathrm{ref}}$ and the true posterior.

### 3.2. Proof of ${\mathrm{ELBO}}_{\mathrm{aux}}\geq {\mathrm{ELBO}}_{\mathrm{init}}$

We prove this by demonstrating that improvement in the ELBO can be guaranteed in our method under the assumption that the conditional variational posterior $q_{\phi_{k-1}}\left(w|a_{k}\right)$ is within the variational family of $q_{\phi_{k}}$, i.e., there exists $\phi_{k}^{*}$, such that $q_{\phi_{k}^{*}}\left(w\right)=q_{\phi_{k-1}}\left(w|a_{k}\right)\propto p\left(a_{k}|w,a_{1:k-1}\right)q_{\phi_{k-1}}\left(w\right)$ for k=1…K.

The central idea is to show that by initializing $\phi_{k}$ at $\phi_{k}^{*}$, the variational distribution remains unchanged—therefore, ${\mathrm{ELBO}}_{\mathrm{aux}}={\mathrm{ELBO}}_{\mathrm{init}}$. Then, as we optimize $\phi_{k}$, we are optimizing the terms in ${\mathrm{ELBO}}_{\mathrm{aux}}$ through $L_{|a_{1:k}}\left(\phi_{k}\right)$. Therefore, ${\mathrm{ELBO}}_{\mathrm{aux}}\geq {\mathrm{ELBO}}_{\mathrm{init}}$.

**Proof.**  We prove ${\mathrm{ELBO}}_{\mathrm{aux}}\geq {\mathrm{ELBO}}_{\mathrm{init}}$ by demonstrating that improvement in the ELBO can be guaranteed in our method under the assumption that the conditional variational posterior $q_{\phi_{k-1}}\left(w|a_{k}\right)$ is within the variational family of $q_{\phi_{k}}\left(w\right)$. i.e.,

(5) $$\forall k\in \left\{1\ldots K\right\}\exists \phi_{k}^{*}\;s.t.\;q_{\phi_{k}^{*}}\left(w\right)=q_{\phi_{k-1}}\left(w|a_{k}\right)\propto p\left(a_{k}|w,a_{1:k-1}\right)q_{\phi_{k-1}}\left(w\right)\;.$$

This assumption holds for all exponential families of distributions.

The objective being optimized in each refinement step is

(6) $$L_{|a_{1:k}}\left(\phi_{k}\right)=E_{q_{\phi_{k}}\left(w\right)}\left[p\left(y|x,w\right)-\log\frac{q_{\phi_{k}}\left(w\right)}{p\left(w|a_{1:k}\right)}\right]\;.$$

From our assumption in Equation (5), it follows that

(7) $$L_{|a_{1:k}}\left(\phi_{k}\right)\geq L_{|a_{1:k}}\left(\phi_{k}^{*}\right)$$

when we reach the global optima $\phi_{k}\leftarrow {arg max}_{\phi_{k}}L_{|a_{1:k}}\left(\phi_{k}\right)$. Even in the case when the optimizer is unable to find the global maximum, it is reasonable to assume that $L_{|a_{1:k}}\left(\phi_{k}\right)\geq L_{|a_{1:k}}\left(\phi_{k}^{*}\right)$, given that we initialize $\phi_{k}$ at $\phi_{k}^{*}$.

The proof is based on mathematical induction on *l*. We show that for l=0…K,

(8) $$\underset{\begin{array}{c} a_{k}∼q_{\phi_{k-1}}\left(a_{k}\right)\\ k=1\ldots l \end{array}}{E}\left[L_{|a_{1:l}}\left(\phi_{l}\right)-\sum_{k=1}^{l}\log\frac{q_{\phi_{k-1}}\left(a_{k}\right)}{p\left(a_{k}|a_{1:k-1}\right)}\right]\geq {\mathrm{ELBO}}_{\mathrm{init}}\;,$$

which holds at l=0, since $L_{|}\left(\phi_{0}\right)={\mathrm{ELBO}}_{\mathrm{init}}$.

Notice that for k=0…K−1,

(9) $$\begin{array}{cc} E_{a_{k+1}∼q_{\phi_{k}}}\left[L_{|a_{1:k+1}}\left(\phi_{k+1}\right)\right]\; & \geq E_{a_{k+1}∼q_{\phi_{k}}}\left[L_{|a_{1:k+1}}\left(\phi_{k+1}^{*}\right)\right]\\ \; & =E_{a_{k+1}∼q_{\phi_{k}}}\left[E_{q_{\phi_{k}}\left(w|a_{k+1}\right)}\left[p\left(y|x,w\right)-\log\frac{q_{\phi_{k}}\left(w|a_{k+1}\right)}{q\left(w|a_{1:k+1}\right)}\right]\right]\\ \; & =E_{a_{k+1}∼q_{\phi_{k}}}\left[E_{q_{\phi_{k}}\left(w|a_{k+1}\right)}\left[p\left(y|x,w\right)-\log\frac{q_{\phi_{k}}\left(w\right)}{p\left(w|a_{1:k}\right)}+\log\frac{q_{\phi_{k}}\left(a_{k+1}\right)}{p\left(a_{k+1}|a_{1:k}\right)}\right]\right]\\ \; & =L_{|a_{1:k}}\left(\phi_{k}\right)+E_{a_{k+1}∼q_{\phi_{k}}}\left[\log\frac{q_{\phi_{k}}\left(a_{k+1}\right)}{q\left(a_{k+1}|a_{1:k}\right)}\right]\;, \end{array}$$

where line 1 follows using Equation (7) and line 3 follows using Bayes’ theorem: $q_{\phi_{k}}\left(w|a_{k+1}\right)$$=\frac{p\left(a_{k+1}|w,a_{1:k}\right)q_{\phi_{k}}\left(w\right)}{q_{\phi_{k}}\left(a_{k+1}\right)}$ and $p\left(w|a_{1:k+1}\right)=\frac{p\left(a_{k+1}|w,a_{1:k}\right)p\left(w|a_{1:k}\right)}{p\left(a_{k+1}|a_{1:k}\right)}$. After rearranging,

(10) $$L_{|a_{1:k}}\left(\phi_{k}\right)\leq E_{a_{k+1}∼q_{\phi_{k}}}\left[L_{|a_{1:k+1}}\left(\phi_{k+1}\right)-\log\frac{q_{\phi_{k}}\left(a_{k+1}\right)}{p\left(a_{k+1}|a_{1:k}\right)}\right]\;.$$

Substituting this into the inductive hypothesis at k=l proves the inductive step as shown next:

(11) $$\begin{array}{cc} \; & {\mathrm{ELBO}}_{\mathrm{init}}\\ \; & \leq \underset{\begin{array}{c} a_{k}∼q_{\phi_{k-1}}\\ k=1\ldots l \end{array}}{E}\left[L_{|a_{1:l}}\left(\phi_{l}\right)-\sum_{k=1}^{l}\log\frac{q_{\phi_{k-1}}\left(a_{k}\right)}{p\left(a_{k}|a_{1:k-1}\right)}\right]\\ \; & \leq \underset{\begin{array}{c} a_{k}∼q_{\phi_{k-1}}\\ k=1\ldots l \end{array}}{E}\left[E_{a_{l+1}∼q_{\phi_{l}}}\left[L_{|a_{1:l+1}}\left(\phi_{l+1}\right)-\log\frac{q_{\phi_{l}}\left(a_{l+1}\right)}{p\left(a_{l+1}|a_{1:l}\right)}\right]-\sum_{k=1}^{l}\log\frac{q_{\phi_{k-1}}\left(a_{k}\right)}{p\left(a_{k}|a_{1:k-1}\right)}\right]\\ \; & =\underset{\begin{array}{c} a_{k}∼q_{\phi_{k-1}}\\ k=1\ldots l+1 \end{array}}{E}\left[L_{|a_{1:l+1}}\left(\phi_{l+1}\right)-\sum_{k=1}^{l+1}\log\frac{q_{\phi_{k-1}}\left(a_{k}\right)}{p\left(a_{k}|a_{1:k-1}\right)}\right] \end{array}$$

To finish the proof, examine the case l=K. Notice that

(12) $$L_{|a_{1:K}}\left(\phi_{K}\right)=E_{q_{\phi_{K}}\left(w\right)}\left[p\left(y|x,w\right)-\frac{q_{\phi_{K}}\left(w\right)}{p\left(w|a_{1:K}\right)}\right]=p\left(y|x,w\right)\;,$$

since $a_{1:K}$ fully determines w, i.e., $q_{\phi_{K}}\left(w\right)=p\left(w|a_{1:K}\right)=\delta_{Dirac}\left(w-\sum_{k=1}^{K}a_{k}\right)$. Substituting Equation (12) in at l=K yields the desired result:

(13) $$\begin{array}{cc} \; & \underset{\begin{array}{c} a_{k}∼q_{\phi_{k-1}}\\ k=1\ldots K \end{array}}{E}\left[L_{|a_{1:K}}\left(\phi_{K}\right)-\sum_{k=1}^{K}\log\frac{q_{\phi_{k-1}}\left(a_{k}\right)}{p\left(a_{k}|a_{1:k-1}\right)}\right]\\ \; & =\underset{\begin{array}{c} a_{k}∼q_{\phi_{k-1}}\\ k=1\ldots K \end{array}}{E}\left[p\left(y|w,x\right)-\sum_{k=1}^{K}\log\frac{q_{\phi_{k-1}}\left(a_{k}\right)}{p\left(a_{k}|a_{1:k-1}\right)}\right]\\ \; & =\underset{q_{\mathrm{ref}}}{E}\left[\log p\left(y|x,w\right)\right]-\mathrm{KL}\left[\;q_{\mathrm{ref}}\left(a_{1:K}\right)\;\;\left|\right|\;\;p\left(a_{1:K}\right)\;\right]\\ \; & ={\mathrm{ELBO}}_{\mathrm{aux}}\geq {\mathrm{ELBO}}_{\mathrm{init}}\;, \end{array}$$

concluding the proof. □

**Figure 4:**
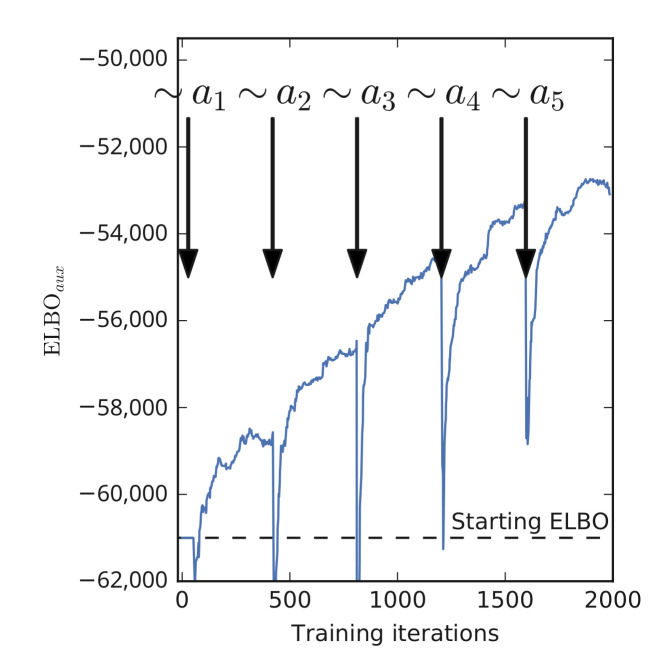
${\mathrm{ELBO}}_{\mathrm{aux}}$ is increasing as we sample the auxiliary variables. Calculated single sample Monte Carlo estimate of the expectation: ${\mathrm{ELBO}}_{\mathrm{aux}}=E_{}\left[\log p\left(y|x,w\right)-\sum_{k=1}^{K}\log\frac{q_{\phi_{k-1}}\left(a_{k}\right)}{p\left(a_{k}|a_{1:k-1}\right)}\right]$ (Equation (13)). The sudden drops after sampling are optimizer artefacts because the momentum is reset after sampling. LeNet-5/CIFAR10.

Note that this result implies that ${\mathrm{ELBO}}_{\mathrm{aux}}$ must grow with each auxiliary variable. We demonstrate this empirically by estimating ${\mathrm{ELBO}}_{\mathrm{aux}}$ as we sample the auxiliary variables in a neural network. The result is shown on Figure 4. We see that ${\mathrm{ELBO}}_{\mathrm{aux}}$ grows after each iteration, exhibiting a stair pattern.

## 4. Experimental Results

We showcase our method on two example tasks: inference in a Bayesian neural network and posterior sampling in a contextual bandit task.

### 4.1. Inference in Deep Neural Networks

The goal of this experiment is twofold. First, we empirically confirm the improvement in the ELBO, and second, we quantify the improvement in the uncertainty estimates due to the refinement. We conduct experiments on regression and classification benchmarks using Bayesian neural networks as the underlying model. We look at the marginal log-likelihood (MLL) of the predictions, as well as accuracy in classification tasks.

We used three baseline models for comparison: mean-field variational inference, multiplicative normalizing flows (MNF), and deep ensemble models. For all methods, we used a batch size of 256 and the Adam optimizer with the default learning rate of 0.001. The hyperparameters of each baseline were tuned using a Bayesian optimization package. We found batch size and learning rate to be consistent across methods.

First, Variational inference (VI, [8,9]). Naturally, we investigate the improvement of our method over variational inference with a mean-field Gaussian posterior approximation. We do inference over all weights and biases with a Gaussian prior centered at 0, the variance of the prior is tuned through empirical Bayes, and the model is trained for 30,000 iterations.

Second, Multiplicative normalizing flows (MNF, [10]). In this work, the posterior means are augmented with a multiplier from a flexible distribution parameterized by the masked RealNVP. This model is trained with the default flow parameters for 30,000 iterations.

Third, Deep ensemble models [11]. Deep ensemble models are shown to be surprisingly effective at quantifying uncertainty. For the regression datasets, we used adversarial training (ϵ=0.01), whereas in classification we did not (since adversarial training did not give an improvement in the classification benchmarks). For each dataset, we trained 10 ensemble members for 5000 iterations each.

Finally, our work, Refined VI. After training the initial mean-field approximation, we generate M=10 refined samples $w_{1:M}$, each with K=5 auxiliary variables. The means on the prior distribution for the auxiliary variables are fixed at 0, and their prior variances form a geometric series (the intuition is that the auxiliary variables carry roughly equal information this way): $\sigma_{a_{k}}^{2}=0.7\left(\sigma_{w}^{2}-\sum_{l=1}^{k-1}\sigma_{a_{l}}^{2}\right)$ for k=1…K. We experimented with different ratios between 0 and 1 for the geometric series and we found that 0.7 worked well. In each refinement iteration, we optimized the posterior with Adam [12] for 200 iterations. To keep the training stable, we kept the learning rate proportional to the standard deviation of the conditional posterior: in iteration *k*, $\mathrm{lr}=0.001\times 0.3^{\frac{k}{2}}$. Our code is available at https://github.com/google/edward2/experimental/auxiliary_sampling.

Following [13], we evaluate the methods on a set of UCI regression benchmarks on a feed forward neural network with a single hidden layer containing 50 units with a ReLU activation function (Table 1). The datasets used a random 80–20 split for training and testing, and we utilize the local reparametrization trick [14].

On these benchmarks, refined VI consistently improves both the ELBO and the MLL estimates over VI. For refined VI, the ${\mathrm{ELBO}}_{\mathrm{ref}}$ cannot be calculated exactly, but ${\mathrm{ELBO}}_{\mathrm{aux}}$ provides a lower bound to it, which we can estimate using Equation (13). Note that the gains in MLL are small in this case. Nevertheless, refined VI is one of the best performing approaches on 7 out of the 9 datasets.

We examine the performance on commonly used image classification benchmarks (Table 2) using LeNet5 architecture [15]. We use the local reparametrization trick [14] for the dense layers and Flipout [16] for the convolutional layers to reduce the gradient noise. We do not use data augmentation in order to stay consistent with the Bayesian framework.

On the classification benchmarks, we again are able to confirm that the refinement step consistently improves both the ELBO and the MLL over VI, with the MLL differences being more significant here than in the previous experiments. Refined VI is unable to outperform deep ensembles in classification accuracy, but it does outperform them in MLL on the largest dataset, CIFAR10.

To demonstrate the performance on larger scale models, we apply the refining algorithm to residual networks [17] with 20 layers (based on Keras’s ResNet implementation). We look at two models: a standard ResNet, where inference is done over every residual block and a hybrid model (ResNet Hybrid [18]), where inference is only done over the final layer of each residual block, and every other layer is treated as a regular layer. For this model, we used a batch-size of 256 and we decayed the learning rate starting from 0.001 over 200 epochs. We used 10 auxiliary variables each reducing the prior variance by a factor of 0.5. Results are shown in Table 3.

Batch normalization [19] provides a substantial improvement for VI though, this improvement interestingly disappears for the hybrid model. The refined hybrid model outperforms the recently proposed natural gradient VI method by [20] in both MLL and accuracy, but it is still behind some non-Bayesian uncertainty estimation methods [21].

### 4.2. Computational Costs

When introducing a novel algorithm for variational inference, we must discuss the computational costs. The computational complexity grows linearly with both *K* and *M*, resulting in an overall O(KM) runtime. The memory requirement is O(M) as it grows linearly with *M*. For the neural network models, the computational cost of generating the posterior samples is ∼30% of the cost of training the initial mean-field approximation (LeNet-5/CIFAR10 on an NVIDIA P100 GPU using TensorFlow). In practice, we recommend tuning the number of auxiliary variables for the given application; using more auxiliary variables always improves the posterior approximation, but they come with additional computational overhead.

### 4.3. Thompson Sampling

Generating posterior samples for Thompson sampling [22,23] in a contextual bandit problem is an ideal use case for the refinement algorithm. Refinement allows one to trade-off computational complexity for a higher quality approximation to the posterior. This can be ideal for Thompson sampling where more expensive objectives often warrant spending time computing better approximations.

Thompson sampling works by sampling a hypothesis from the approximate posterior to decide on each action. This balances exploration and exploitation, since probable hypotheses are tested more frequently than improbable ones. In each step,

1. Sample $w∼q_{\phi }\left(w\right)$;
2. Take action ${arg max}_{a}E_{p(r|c,a,w)}\left[r\right]$, where *r* is the reward that is determined by the context *c*, the action *a* taken, and the unobserved model parameters w;
3. Observe reward *r* and update the approximate posterior $q_{\phi }\left(w\right)$.

We look at the mushroom task [9,24], where the agent is presented with a number of mushrooms that they can choose to eat or pass. The mushrooms are either edible or poisonous. Eating an edible mushroom always yield a reward of 5, while eating a poisonous mushroom yield a reward 5 with probability 50% and −35 with probability 50%. Passing a mushroom gives no reward.

To predict the distribution of the rewards, the agent uses a neural network with 23 inputs and two outputs. The inputs are the 22 observed attributes of the mushrooms and the proposed action (1 for eating and 0 for passing). The output is the mean expected reward. The network has a standard feed-forward architecture with two hidden layers containing 100 hidden units each, with ReLU activations throughout. For the prior, we used a standard Gaussian distribution over the weights.

For the variational posterior, we use a mean-field Gaussian approximation that we update for 500 iterations after observing each new reward. For the updates, we use batches of 64 randomly sampled rewards with an Adam optimizer with learning rate $10^{-3}$. In refined sampling, we used two auxiliary variables: $w=a_{1}+a_{2}$ with $p\left(a_{1}\right)=N\left(0,0.7\right)$ and $p\left(a_{2}\right)=N\left(0,0.3\right)$. To obtain a high quality sample for prediction, we first draw $a_{1}$ using the main variational approximation and then refine the posterior over $a_{2}$ for 500 iterations. After using the refined sample for prediction, we discard it and update the main variational approximation using the newly observed reward (for 500 iterations). In our experiments, we used three posterior samples to calculate the expected reward, which helps to emphasize exploitation compared to using a single sample.

As baselines, we show the commonly used ϵ-greedy algorithm, where the agent takes the action with the highest expected reward according to the maximum-likelihood solution with probability 1−ϵ, and takes a random action with probability ϵ.

**Figure 5:**
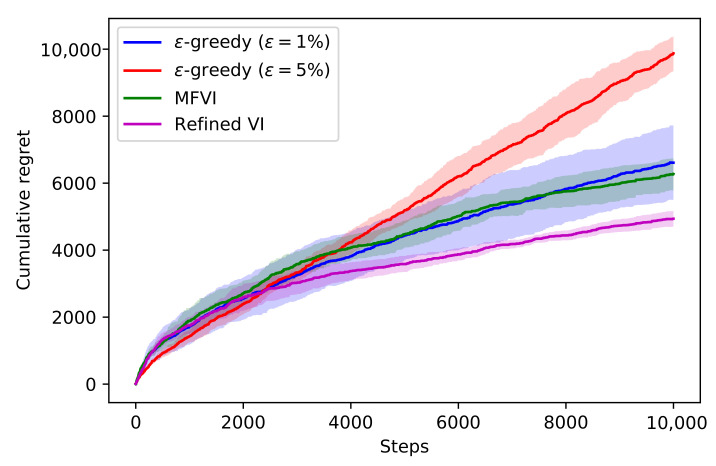
The performances of ϵ-greedy, Mean-field VI, and Refined VI on the mushrooms contextual bandit task. Lower regret is better. The mean and standard deviations are shown from 5 runs with different random seeds.

We measure the performance using the cumulative regret. The cumulative regret measures the difference between our agent and an omniscient agent that makes the optimal choice each time. Lower regret indicates better performance. Figure 5 depicts the results. We see that the refined agent has lower regret throughout, which shows that the higher quality posterior samples translate to improved performance. Until about 3000 iterations, the ϵ-greedy algorithms perform well, but they are overtaken by Thompson sampling as the posterior tightens and the agent shifts focus to exploitation.

## 5. Related Works

Although, in theory, the Bayesian approach can accurately capture uncertainty, in practice, we find that exact inference is computationally infeasible in most scenarios, and thus, we have to resort to approximate inference methods. There is a wealth of research on approximate inference methods; here, we focus on works closely related to this paper.

Variational inference [25] tries to approximate the true posterior distribution over parameters with a variational posterior from a simple family of distributions. Mean-field VI, which for neural networks traces back to [26], uses independent Gaussian distributions over the parameters to try to capture the posterior. The advantage of the mean-field approximation is that the network can be efficiently trained using the reparameterization trick [27], and the variational posterior has a proper density over the parameter space, which then can be used across tasks, such as continual learning [20,28] and contextual bandits [29]. Recently, [10] showed that normalizing flows can be used to further increase the flexibility of the variational posterior. [30] provide a detailed survey of recent advances in VI.

Our method is a novel variant of the auxiliary variable approaches to VI [31,32] that increase the flexibility of the variational posterior through the use of auxiliary variables. The key distinction, however, is that instead of trying to train a complex variational approximation over the joint distribution, we iteratively train simple mean-field approximations at the sampled values of the auxiliary variables. Although this poses an O(MK) overhead (*K* is the number of auxiliary variables and *M* is the number of posterior samples) over mean-field VI, the training itself remains straightforward and efficient. The introduction of every new auxiliary variable increases the flexibility of the posterior approximation. In contrast to MCMC methods, it is unclear whether the algorithm approaches the true posterior in the limit of infinitely many auxiliary variables.

There are also numerous methods that start with an initial variational approximation and refine it through a few MCMC steps [33,34,35]. The distinction from our algorithm is that we refine the posterior starting at large scale and iteratively move towards smaller scale refinements, whereas these methods only refine the posterior at the scale of the MCMC steps [36,37,38] used boosting to refine the variational posterior, where they iteratively added parameters, such as mixture components to minimize the residual of the ELBO. Our method does not add parameters at training time but instead iteratively refines the samples through the introduction of auxiliary variables. We do not include these in our baselines since they have yet to be applied to Bayesian multi-layer neural networks.

Further related works include methods that iteratively refine the posterior in latent variable models [39,40,41,42]. These methods, however, focus on reducing the amortization gap and do not increase the flexibility of the variational approximation.

Lastly, there are non-Bayesian strategies for estimating epistemic uncertainty in deep learning. Bootstrapping [43] and deep ensembles [11] may be the most promising. Deep ensembles, in particular, have been demonstrated to achieve strong performance on benchmark regression and classification problems and uncertainty benchmarks including out-of-distribution detection [11] and prediction under distribution shift [18]. Both methods rely on constructing a set of independently trained models to estimate the uncertainty. Intuitively, the amount of disagreement across models reflects the uncertainty in the ensemble prediction. In order to induce diversity among the ensemble members, bootstrapping subsamples the training set for each member while deep ensembles use the randomness in weight initialization and mini-batch sampling.

## 6. Conclusions

In this work, we investigated a novel method for generating samples from a highly flexible posterior approximation, which works by starting with a mean-field approximation and locally refining it in selected regions. We demonstrated that the samples are able to capture dependencies and multi-modality. Furthermore, we showed both theoretically and empirically that the method always improves the ELBO of the initial mean-field approximation and demonstrated its improvement on a hierarchical inference problem, a deep learning benchmark and a contextual bandit task.

## Figures and Tables

**Table 1:** Refining improves the ELBO across all regression benchmarks. Results on the UCI regression benchmarks with a single hidden layer containing 50 units. Metrics: marginal log-likelihood (MLL, higher is better), and the evidence lower bound (ELBO higher is better). The mean values and standard deviations are shown in the table. Bolded numbers indicate the highest ELBO (${\mathrm{ELBO}}_{\mathrm{aux}}$ is a lower bound to ${\mathrm{ELBO}}_{\mathrm{ref}}$, which is the true ELBO) and underlined numbers indicate the highest MLL.

	Deep Ensemble	MNF	VI		Refined VI (This Work)	
	MLL	MLL	MLL	ELBO	MLL	${\mathrm{ELBO}}_{\mathrm{aux}}$
Boston	−9.136 ± 5.719	−2.920 ± 0.133	−2.874 ± 0.151	−668.2 ± 7.6	−2.851 ± 0.185	**−630.3** ± 7.7
Concrete	−4.062 ± 0.130	−3.202 ± 0.055	−3.138 ± 0.063	−3248.1 ± 68.5	−3.131 ± 0.062	**−3071.1** ± 64.0
Naval	3.995 ± 0.013	3.473 ± 0.007	5.969 ± 0.245	53,440.7 ± 2047.3	6.128 ± 0.171	**54,882.6** ± 1228.3
Energy	−0.666 ± 0.058	−0.756 ± 0.054	−0.749 ± 0.068	−1296.7 ± 66.3	−0.707 ± 0.094	**−1192.3** ± 62.0
Yacht	−0.984 ± 0.104	−1.339 ± 0.170	−1.749 ± 0.232	−928.7 ± 112.9	−1.626 ± 0.231	**−790.0** ± 84.7
Kin8nm	1.135 ± 0.012	1.125 ± 0.022	1.066 ± 0.019	6071.2 ± 61.7	1.069 ± 0.018	**6172.7** ± 67.6
Power	−3.935 ± 0.140	−2.835 ± 0.033	−2.826 ± 0.020	−22,496.5 ± 130.4	−2.820 ± 0.024	**−22,368.9** ± 85.3
Protein	−3.687 ± 0.013	−2.928 ± 0.0	−2.926 ± 0.010	−108,806.007 ± 174.5	−2.923 ± 0.009	**−108,597.5** ± 158.4
Wine	−0.968 ± 0.079	−0.963 ± 0.027	−0.973 ± 0.054	−1346.1 ± 18.0	−0.968 ± 0.056	**−1311.8** ± 17.4

**Table 2:** Refining improves the ELBO across all image classification benchmarks. Results on image classification benchmarks with the LeNet-5 architecture, *without data augmentation*. Metrics: marginal log-likelihood (MLL, higher is better), accuracy (Acc, higher is better), and the evidence lower bound (ELBO higher is better). Means and standard deviations are shown. Bolded numbers indicate the highest ELBO (${\mathrm{ELBO}}_{\mathrm{aux}}$ is a lower bound to ${\mathrm{ELBO}}_{\mathrm{ref}}$, which is the true ELBO) and underlined numbers indicate the highest MLL.

	Deep Ensemble	MNF	VI		Refined VI (This Work)	
	MLL & Acc	MLL & Acc	MLL & Acc	ELBO	MLL & Acc	${\mathrm{ELBO}}_{\mathrm{aux}}$
mnist	−0.017 ± 0.001	−0.034 ± 0.002	−0.032 ± 0.001	−7618.5 ± 47.5	−0.025 ± 0.001	**−6310.8** ± 42.3
	99.4% ± 0.0	99.1% ± 0.1	99.1% ± 0.1		99.2% ± 0.0	
fashion_mnist	−0.201 ± 0.002	−0.255 ± 0.004	−0.255 ± 0.003	−22,830.3 ± 232.6	−0.241 ± 0.004	**−20,438.9** ± 79.6
	93.1% ± 0.1	90.7% ± 0.2	90.7% ± 0.1		91.3% ± 0.2	
cifar10	−0.791 ± 0.009	−0.795 ± 0.013	−0.815 ± 0.004	−57,257.8 ± 299.5	−0.768 ± 0.007	**−50,989.2** ± 238.9
	76.3% ± 0.3	72.8% ± 0.6	72.3% ± 0.5		73.5% ± 0.5	

**Table 3:** Results on CIFAR10 with the ResNet architecture, *without data augmentation*. We observe that our method not only improves significantly in MLL over the VI baseline, but it also significantly improves in accuracy over the strong ensemble baseline. Metrics: marginal log-likelihood (MLL, higher is better), accuracy (Acc, higher is better), and the evidence lower bound (ELBO higher is better). Note that the non-hybrid and the hybrid models are equivalent when trained deterministically. The best MLL result is highlighted in bold.

	Deep Ensemble		VI		Refined VI (This Work)	
	MLL	Acc	MLL	Acc	MLL	Acc
ResNet	−0.698	82.7%	−0.795	72.6%	**−0.696**	75.5%
ResNet + BatchNorm	**−0.561**	83.6%	−0.672	77.6%	−0.593	79.7%
ResNet Hybrid	−0.698	82.7%	−0.465	84.2%	**−0.432**	85.8%
ResNet Hybrid + BatchNorm	−0.561	83.6%	−0.465	84.0%	**−0.423**	85.6%

## Appendix A. Analytical Forms of *q*_*ϕ*_*k*−1__ (a_*k*_) and *q*_*ϕ*_*k*−1__ (w|a_*k*_)

For a diagonal Gaussian prior distribution $p\left(w\right)=N\left(w|0,\sigma^{2}I\right)$ (0 denotes the $\;d_{w}$ dimensional zero vector and *I* denotes the $\;d_{w}\times \;d_{w}$ identity matrix where $\;d_{w}$ is the dimensionality of w), we have $w=\sum_{k=1}^{K}a_{k}$, $p\left(a_{k}\right)=N\left(a_{k}|0,\sigma_{k}^{2}I\right)$ for k∈{1,…,K} such that $\sum_{k=1}^{K}\sigma_{k}^{2}=\sigma^{2}\;$.

The forms of approximate posterior over the auxiliary variables $q_{\phi_{k-1}}\left(a_{k}\right)$ and the conditionals $q_{\phi_{k-1}}\left(w|a_{k}\right)$ can be computed in closed form. We only derive the result in the univariate case, but extending to the diagonal covariance case is straightforward.

First, let $p\left(a_{k}\right)=N\left(a_{k}\;|\;{µ}_{k},\sigma_{k}^{2}\right)$. Now, define $b_{k}=\sum_{i=1}^{k}a_{i}$, $m_{k}=\sum_{i=k+1}^{K}{µ}_{i}$ and $s_{k}^{2}=\sum_{i=k+1}^{K}\sigma_{i}^{2}$. Since $z=\sum_{k=1}^{K}a_{k}$, using the formula for the conditional distribution of sums of Gaussian random variables (For Gaussian random variables X,Y with means ${µ}_{x},{µ}_{y}$ and variances $\sigma_{x}^{2},\sigma_{y}^{2}$ and Z=X+Y, p(x|z) is normally distributed with mean ${µ}_{x}+\left(z-{µ}_{x}-{µ}_{y}\right)\frac{\sigma_{x}^{2}}{\sigma_{x}^{2}+\sigma_{y}^{2}}$ and variance $\frac{\sigma_{x}^{2}\sigma_{y}^{2}}{\sigma_{x}^{2}+\sigma_{y}^{2}}$), we obtain

(A1) $$p\left(a_{k}\;|\;a_{1:k-1},w\right)=N\left(a_{k}|{µ}_{k}+\left(w-b_{k-1}-m_{k-1}\right)\frac{\sigma_{k}^{2}}{s_{k-1}^{2}},\frac{s_{k}^{2}\sigma_{k}^{2}}{s_{k-1}^{2}}\right).$$

Recall that

(A2) $$q_{\phi_{k-1}}\left(a_{k}\right)=\int p\left(a_{k}|a_{1:k-1},w\right)q_{\phi_{k-1}}\left(w\right)\;dw\;,$$

and assume that we have already calculated $q_{\phi_{k-1}}\left(w\right)=N\left(w\;|\;\nu_{k-1},\rho_{k-1}^{2}\right)$. Notice that the quantity of interest is an integral of Gaussian densities, and hence after some algebraic manipulation, we obtain

(A3) $$q_{\phi_{k-1}}\left(a_{k}\right)=N\left(a_{k}|{µ}_{k}+\left(\nu_{k-1}-b_{k-1}-m_{k-1}\right)\frac{\sigma_{k}^{2}}{s_{k-1}^{2}},\frac{s_{k}^{2}\sigma_{k}^{2}}{s_{k-1}^{2}}+\rho_{k-1}^{2}\frac{\sigma_{k}^{4}}{s_{k-1}^{4}}\right)\;.$$

Regarding $q_{\phi_{k-1}}\left(w|a_{k}\right)$, we have

(A4) $$q_{\phi_{k-1}}\left(w|a_{k}\right)=\frac{p\left(a_{k}\;|\;a_{1:k-1},w\right)q_{\phi_{k-1}}\left(w\right)}{q_{\phi_{k-1}}\left(a_{k}\right)}\;$$

using Bayes’ rule. Again, we see that the desired quantity is a product of Gaussians, which we can derive to arrive at

(A5) $$q_{\phi_{k-1}}\left(w|a_{k}\right)=N\left(w|\frac{\left(a_{k}-{µ}_{k}\right)\rho_{k-1}^{2}s_{k-1}^{2}+\left(b_{k-1}+m_{k-1}\right)\sigma_{k}^{2}\rho_{k-1}^{2}+\nu_{k-1}s_{k}^{2}s_{k-1}^{2}}{\sigma_{k}^{2}\rho_{k-1}^{2}+s_{k-1}^{2}s_{k}^{2}},\frac{\rho_{k-1}^{2}s_{k-1}^{2}s_{k}^{2}}{\sigma_{k}^{2}\rho_{k-1}^{2}+s_{k-1}^{2}s_{k}^{2}}\right)\;.$$
